# Tumor derived stress triggers C/EBPβ homologous protein (Chop) expression in myeloid derived suppressor cells (MDSC) and mediates immunosuppressive activity

**DOI:** 10.1186/2051-1426-2-S3-O16

**Published:** 2014-11-06

**Authors:** Paul Thevenot, Rosa Sierra, Patrick Raber, Paulo Rodriguez

**Affiliations:** 1 Stanley S. Scott Cancer Center, Louisiana State University Health Sciences Center, New Orleans, LA, USA

## 

Suppression of anti-tumor T cell responses by MDSC remains a significant barrier in cancer immunotherapy. Although several pathways have been characterized as critical for MDSC-induced suppression, there are currently no therapies to globally and specifically inhibit MDSC function. We postulate that identifying and inhibiting the central mediators of MDSC-regulatory activity will overcome T cell suppression and increase the efficacy of T cell-based immunotherapy in cancer. We aimed to determine the role of the common stress sensor C/EBP-homologous-stress-related protein (Chop), a downstream product of integrated stress responses, as a master regulator of MDSC-suppressive activity. Our results show that Chop is preferentially expressed in malignant cells and MDSC in s.c. mouse tumors. Selective expression of Chop was also detected in tumor-infiltrating MDSC from colon carcinoma patients. Interestingly, injection of tumor cells having functional Chop into systemic Chop -/- mice or Chop null bone marrow chimeric mice resulted in a significant anti-tumor effect mediated by CD8^+ ^T cells, suggesting the importance of MDSC-Chop in tumor-induced tolerance. In fact, deletion of Chop in MDSC increased the efficacy of T cell-based immunotherapy. MDSC isolated from tumor-bearing Chop null mice had decreased ability to block T cell responses; impaired expression of major MDSC-inhibitory pathways; and a surprising ability to prime T cell proliferation and induce anti-tumor effects. Accordingly, depletion of Gr-1^+ ^MDSC restored tumor growth in Chop -/- mice, while it prevented tumor growth in wild type mice, confirming functional differences in MDSC from wild type and Chop -/- mice. To therapeutically block Chop in tumors, we used a specific liposomal-encapsulated siRNA, which successfully blocked Chop expression and induced anti-tumor effects. We next examined the effects of Chop on C/EBPβ and STAT-3, both master regulators of MDSC function. MDSC from Chop -/- mice had elevated expression of C/EBPβ inhibitory isoform LIP, low C/EBPβ binding to IL-6-promoter, decreased IL-6 production, and impaired expression of IL-6 target phospho-STAT-3. Also, Chop -/- MDSC expressed higher levels of miR-142-3p, a mi-RNA that promotes C/EBPβ LIP over LAP and LAP*. Ectopic expression of IL-6 in tumors restored tumor growth, MDSC suppression, and C/EBPβ and phospho-STAT-3 levels in Chop -/- mice, suggesting the role of this pathway in the effects induced by Chop deletion. Collectively, this data suggests the role of Chop as a master regulator of the immune inhibitory activity of MDSC and justify the potential targeting of Chop as a way to restore protective immunity in cancer.

## Consent

Written informed consent was obtained from the patient for publication of this abstract and any accompanying images. A copy of the written consent is available for review by the Editor of this journal.

